# Sex differences in medico-legal action against doctors: a systematic review and meta-analysis

**DOI:** 10.1186/s12916-015-0413-5

**Published:** 2015-08-13

**Authors:** Emily Unwin, Katherine Woolf, Clare Wadlow, Henry W. W. Potts, Jane Dacre

**Affiliations:** UCL Medical School, University College London, Royal Free Hospital, London, NW3 2PF UK; Institute of Health Informatics, University College London, 222 Euston Road, London, NW1 2DA UK; Royal College of Physicians, 11 St Andrews Place, London, NW1 4LE UK

**Keywords:** Disciplinary action, Malpractice, Medical education, Medical regulatory boards, Meta-analysis, Systematic review

## Abstract

**Background:**

The relationship between male sex and poor performance in doctors remains unclear, with high profile studies showing conflicting results. Nevertheless, it is an important first step towards understanding the causes of poor performance in doctors. This article aims to establish the robustness of the association between male sex and poor performance in doctors, internationally and over time.

**Methods:**

The electronic databases MEDLINE, EMBASE, and PsycINFO were searched from inception to January 2015. Backward and forward citation searching was performed. Journals that yielded the majority of the eligible articles and journals in the medical education field were electronically searched, along with the conference and poster abstracts from two of the largest international medical education conferences. Studies reporting original data, written in English or French, examining the association between sex and medico-legal action against doctors were included. Two reviewers independently extracted study characteristics and outcome data from the full texts of the studies meeting the eligibility criteria. Study quality was assessed using the Newcastle-Ottawa scale. A random effect meta-analysis model was used to summarize and assess the effect of doctors’ sex on medico-legal action. Extracted outcomes included disciplinary action by a medical regulatory board, malpractice experience, referral to a medical regulatory body, complaints received by a healthcare complaints body, criminal cases, and medico-legal matter with a medical defence organisation.

**Results:**

Overall, 32 reports examining the association between doctors’ sex and medico-legal action were included in the systematic review (n=4,054,551), of which 27 found that male doctors were more likely to have experienced medico-legal action. 19 reports were included in the meta-analysis (n=3,794,486, including 20,666 cases). Results showed male doctors had nearly two and a half times the odds of being subject to medico-legal action than female doctors. Heterogeneity was present in all meta-analyses.

**Conclusion:**

Male doctors are more likely to have had experienced medico-legal actions compared to female doctors. This finding is robust internationally, across outcomes of varying severity, and over time.

**Electronic supplementary material:**

The online version of this article (doi:10.1186/s12916-015-0413-5) contains supplementary material, which is available to authorized users.

## Background

Between 2010 and 2013, there was a 64 % rise in the number of complaints to the General Medical Council (GMC) – the United Kingdom (UK) medical regulator, about doctors’ fitness to practise [1]. This period also saw a 42 % rise in the number of doctors prevented from practising medicine through erasure or suspension from the UK medical register [1]. Similarly, in the United States, the number of state board disciplinary actions increased between 2008 and 2012, with a 17 % increase in the number of medical licenses that were revoked, denied, or suspended [2].

The burden of investigating complaints about doctors’ fitness to practise not only places an enormous level of stress on the doctor being investigated, as highlighted by a recent report on the impact on the mental well-being of doctors undergoing investigation [3], but also places a resource strain on regulators [4]. The increase in the number of investigations may also lead to patient concerns about the quality of care they receive. The identification of predictors of disciplinary action is an important step toward aiding the medical profession, medical regulators, and medical educationalists to understand the underlying causal factors, better support doctors in achieving the standards expected of them, and improve patient care.

A study of all registered doctors in the UK in 2013 demonstrated that the sex of a doctor was an important factor associated with disciplinary action by the GMC [5]. Female doctors were less likely to receive disciplinary action, even after taking into account other explanatory variables such as years since qualification and specialty. That study provided a snapshot of the situation in the UK, but did not include doctors practising outside the UK, nor did it include other measures of poor performance not resulting in disciplinary action by the GMC but nonetheless serious. Several studies examining sex differences in disciplinary action against doctors across the world have been conducted, and the results of these studies vary– some conclude that male doctors are more likely to be disciplined [6, 7], but with varying effect sizes, while others have not found a significant association [8, 9]. It is not clear whether sex differences are robust across contexts and across measures of performance. Establishing the generalizability and an overall effect size internationally, over time, and on multiple measures of poor performance, will help us to understand what factors result in poor professional performance and how to remediate it.

In the present study we completed a systematic review of the literature and meta-analysis to answer the following questions: (1) Was the sex difference observed in UK doctors in 2013 also present in different countries, with different medical systems and cultures? (2) Has the sex difference varied over the last four decades? (3) Are sex differences present on measures of poor performance other than disciplinary action, such as malpractice litigation?

## Methods

We used guidance published by PRISMA [10] and Cochrane [11] to guide our methodology.

### Data sources and search strategies

We conducted systematic searches (from inception to January 2015) of MEDLINE, Embase, and PsycINFO for studies describing the association between doctors’ sex and experience of medico-legal action (Additional file 1: Table S1 for the search terms used). In addition, we performed backward and forward citation searching and searched electronically within the journals that yielded the majority of the eligible articles, along with journals important in the medical education field, for relevant articles. Finally, we electronically searched the conference and poster abstracts published from two of the largest international medical education conferences for relevant literature (Additional file 1: Table S2 for journal/conference titles). Studies not published in English or French were excluded due to limited resources.

### Study selection

One researcher (EU) assessed the eligibility of identified studies, without consideration of their results. Articles were considered for inclusion in the systematic review if (1) the study included data from an original and peer-reviewed study, (2) the study participants were medical doctors, (3) the authors reported an effect estimate (or provided data that enabled the calculation of an effect estimate) or the proportion of male and female participants who had experienced a medico-legal action.

We considered all studies, regardless of study design, and we used broad criteria to define the outcome term medico-legal action (see below for the definition of each outcome term used). We identified articles eligible for further review by performing an initial screen of titles and/or abstracts, followed by a full-text review.

### Data extraction

Two researchers (EU and CW) independently extracted data from all the eligible studies using a pre-determined data abstraction form modified from the Cochrane Handbook [11]. Each researcher independently recorded information on study characteristics (authors, publication year, journal, country, study design, years study conducted, sampling method, data collection method), participants’ characteristics (sex, specialty, grade, number), method by which exposure data was collected, and main outcome (type, method by which data was collected). We also recorded information on analysis strategy and reported proportion of outcome for each sex, or odds ratio with confidence intervals. When reports contained multivariate analyses, we prioritised crude effects; however, if no crude effects were reported, we included outcome measures adjusted for other variables. We assessed the methodological quality of each of the studies using the Newcastle-Ottawa scale [12]. Any discrepancies in the data extraction process were reconciled through discussion.

### Outcome definition and subgroup analyses

We used a variety of outcome definitions in an attempt to capture as much literature as possible and allow for the variety of terms to describe medico-legal actions used by different countries. Two researchers (EU and CW) independently selected the most relevant outcome definition for each study included in the review. Any disagreement about outcome category was resolved through consensus. Throughout this report, we have used the term ‘medico-legal action’ to group together and represent all of the outcome types.

#### Disciplinary action by a medical regulatory body

Disciplinary action taken against the doctor by a medical regulatory board.

#### Malpractice experience

Malpractice claims and malpractice cases.

#### Complaints received by a medical regulatory body

Complaints or referrals received by a medical regulatory body about a doctor’s practice.

#### Complaints received by a healthcare complaints body

Complaints received by an organisation other than a medical regulatory body, whose function is to help investigate health care complaints and provide advice on how to handle the case.

#### Criminal case

Any sanctions imposed by the criminal justice system for criminal activities performed while practising as a doctor.

#### Medico-legal matter with a medical defence organisation

This umbrella term was used when a study grouped together and examined several outcome types and it was not possible to examine each outcome type separately. The included outcomes were malpractice claim, complaint to a healthcare complaints body or medical regulatory body, disciplinary hearing by a medical regulatory body, and criminal charges, among others.

We decided, *a priori*, to perform subgroup analyses based on study design, country where the study population were employed, type of outcome measure, grade and specialty of the doctors within the study population, and the most recent year in which the data was collected (if missing, year of publication was used). We chose those variables because of their potential impact on any association between doctors’ sex and medico-legal action: study design can influence the types of bias introduced; medical and legal systems vary between countries, and complaints may be dealt with differently in different systems; the proportion of women practising medicine has been increasing over time; and specialty and grade of a doctor have been demonstrated to be associated with medico-legal action [5].

### Statistical analysis

We performed the main analysis for all the studies combined. We then conducted subgroup analyses on variables selected *a priori*. To ensure there were sufficient studies in each stratum to demonstrate a meaningful result, the outcome variable was grouped into three categories: ‘Disciplinary action’, ‘Malpractice’, and ‘Other’. The variable ‘country’ was grouped into three categories that represented the continents from which the studies arose: ‘North America’, ‘Asia and Australia’, and ‘Europe’. The year variable was grouped into six 5-year bands (1985–89, 1990–94, 1995–99, 2000–04, 2005–09, and 2010–14).

We calculated an estimate for each study for the effect of male sex on medico-legal action, and performed heterogeneity tests. We then calculated a summary estimate of effect of male sex on experience of medico-legal action using the random-effects model. Meta-analyses followed to enable the provision of statistical evidence of heterogeneity.

## Results

### Systematic review

**Fig. 1:**
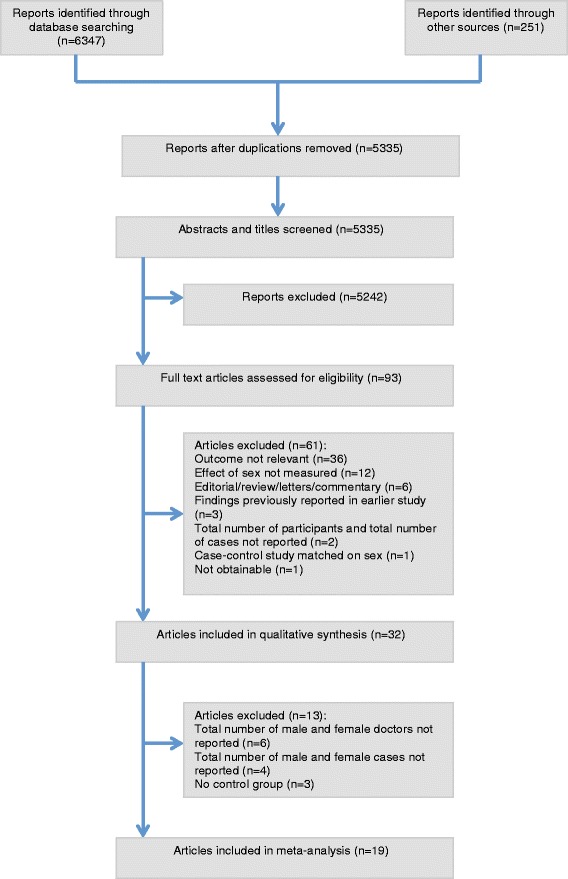
Flow chart showing reports retrieved, and articles excluded and included in the review based on the PRISMA Statement [10]

We retrieved 6,598 citations, of which 32 studies met the inclusion criteria for the systematic review (Fig. 1). A study population (including both cases and non-cases) of 4,054,551 was captured by the included studies, of which over 40,246 are cases of medico-legal actions. Study characteristics are shown in Table 1.

**Table 1:** Characteristics of eligible studies

First author, year (country)	Study design	Description of study population	Years data collected	Data source	Outcomes assessed	Statistical test
Alam et al. [13], 2013 (Canada)	Cohort	Anaesthetists of all grades	2000–2011	Medical regulatory authority (College of Physicians and Surgeons)	Disciplinary action	Proportion
Alam et al. [14], 2011 (Canada)	Cohort	Doctors of all specialties and grades	2002–2009	Medical regulatory authority (College of Physicians and Surgeons)	Disciplinary action	Proportion
Balch et al. [23], 2011 (USA)	Cross-sectional	Surgeons of all grades	2010	Electronic questionnaire	Malpractice suit in last 2 years	χ^2^ *P* <0.01
Baldwin et al. [24], 1991 (USA)	Cohort	General practitioners and obstetricians of all grades	1982–1988	Insurance company	Malpractice experience	χ^2^ *P* >0.05
Birkeland et al. [15], 2013 (Denmark)	Cohort	General practitioners; grades of doctors not stated	2007	Medical regulatory authority (Complaint handling authority)	Disciplinary action	Multivariate analysis adjusted for complaint motives, patient characteristics, GP characteristics
Bismark et al. [38], 2013 (Australia)	Cohort	All specialties of all grades	2000–2011	Health Service Commissions	Patient complaints	Proportion
Cardarelli et al. [16], 2004 (USA)	Case–control	All specialties of all grades	1989–1998	Medical regulatory authority (State Board of Medical Examiners)	Disciplinary action	Multivariate analysis adjusted for years in practice, ethnicity, international education, specialty, method of licensure
Chauhan et al. [25], 2005 (USA)	Cross-sectional	Obstetricians and gynaecologists, excluded residents	Not reported	Postal questionnaire	Malpractice claim	Multivariate analysis adjusted for age, ethnicity, years in practice, no subspecialty
Clay et al. [17], 2003 (USA)	Case–control	Majority of specialties ^a^ of all grades	1997–1999	Medical regulatory authority (State Medical Board)	Disciplinary action	Univariate analysis with controls matched on location
Donaldson et al. [39], 2014 (England)	Cohort	All specialties of all grades	2001–2012	National Clinical Assessment Service	Referral to National Clinical Assessment Service	Univariate analysis
Elkin et al. [18], 2011 (Australia & New Zealand)	Cohort	All specialties of all grades	2000–2009	Written determinations	Disciplinary action	Rate
Ely et al. [26], 1999 (USA)	Cohort	General practitioners, excluding doctors who were unlicensed or recently licensed	1971–1994	Insurance company	Malpractice claims	Univariate and multivariate analysis adjusted for international education, board certification, physician’s recognition award, practice location
Goldenbaum et al. [36], 2008 (USA)	Cohort	All specialties of all grades	1998–2006	Online and published sources and databases	Criminal and administrative cases involving controlled substances	χ^2^ *P* <0.001
Hickson et al. [27], 2002 (USA)	Cohort	Majority of specialties ^b^ excluding residents	1992–1998	Patient Advocates Office and Office of Insurance and Risk Management	Malpractice (at least one lawsuit)	χ^2^ *P* <0.001
Khaliq et al. [19], 2005 (USA)	Cohort	All specialties of all grades	2001	Medical regulatory authority (State Medical Board)	Disciplinary action	Univariate and multivariate analysis adjusted for ethnicity, board certification, international education, specialty
Kohatsu et al. [6], 2004 (USA)	Case–control	All specialties of all grades	1998–2001	Medical regulatory authority (State Medical Board) and American Medical Association e-Physician Profiles system	Disciplinary action	Univariate and multivariate analysis adjusted for age, board certification, international education, specialty
Morrison et al. [7], 1998 (USA)	Case–control	All specialties of all grades	1995–1997	Medical regulatory authority (State Medical Board) and Directory of Physicians in the United States	Disciplinary action	Univariate analysis
Nash et al. [35], 2009 (Australia)	Cross-sectional	All specialties of all grades	2007	Postal questionnaire	Medico-legal matter	Univariate and multivariate analysis adjusted for age, marital status, specialty, international education, solo practice, hours worked per week, peer review in past 12 months, CME requirements, teaching role, AUDIT score, GHQ score
Nash et al. [34], 2009 (Australia)	Cross-sectional	General practitioners; grades of doctors not stated	2006	Postal questionnaire and insurance company	Medico-legal matter	χ^2^ *P* <0.001
Pande et al. [37], 2013 (USA)	Cohort	All specialties of all grades	2000–2011	Office of the Inspector General of the US Department of Health and Human Services	Criminal case (convicted of Medicare and Medicaid fraud)	Proportion
Papadakis et al. [8], 2004 (USA)	Case–control	All specialties of all grades	1990–2000	Medical regulatory authority (State Medical Board)	Disciplinary action	Univariate and multivariate analysis adjusted for undergraduate GPA, MCAT score, did not pass medical school course, professionalism severity ranking
Papadakis et al. [9], 2005 (USA)	Case–control	All specialties of all grades	2990–2003	Medical regulatory authorities (Federation of State Medical Boards)	Disciplinary action	Univariate and multivariate analysis adjusted for MCAT score, number of medical school courses not passed, unprofessional behaviour in medical school
Papadakis et al. [20], 2008 (USA)	Cohort	Internal medicine residents	2000–2006	American Board of Internal Medicine	Disciplinary action	Univariate and multivariate analysis adjusted for performance during residency, international education, no subspecialty certification
St George [32], 2003 (New Zealand)	Cohort	All specialties of all grades	1996–2002	Medical regulatory authority (Medical Council)	Referral to medical regulatory body	Proportion
Tamblyn et al. [33], 2007 (Canada)	Cohort	All specialties of all grades	1993–1996	Medical regulatory authorities	Referral to medical regulatory body	Univariate and multivariate analysis adjusted for examination score, international education, specialty, practice location
Taragin et al. [28], 1992 (USA)	Cohort	All specialties, excluding <2 years of observations	1977–1987	Insurance company	Malpractice claims	Multivariate analysis adjusted for medical degree type, international education, board certification
Unwin et al. [5], 2014 (UK)	Cross-sectional	All specialties of all grades	2013	Medical regulatory authority (Medical Council)	Disciplinary action	Univariate and multivariate analysis adjusted for years since qualification, international education, specialty
Wakeford [21], 2011 (UK)	Cross-sectional	All specialties of all grades	2011	Medical regulatory authority (Medical Council)	Disciplinary action	χ^2^ *P* <0.001
Weisman et al. [29], 1988 (USA)	Cross-sectional	Obstetricians and gynaecologists excluding residents	1984	Postal questionnaire and telephone survey	Malpractice litigation	Multivariate analysis adjusted for practice type and location, years since residency, board certification, work type, patient demographics, international education
Weycker et al. [30], 2000 (USA)	Cohort	All specialties of all grades	1980–1989	Insurance company and American Medical Association Physician Masterfiles	Malpractice claims	Multivariate analysis adjusted for prior claims, educational characteristics, demographic characteristics, practice characteristics
Wu et al. [31], 2009 (Taiwan)	Cross-sectional	All specialties of all grades	1991and 2005	Postal questionnaire	Malpractice claims	Multivariate analysis adjusted for age, specialty
Yates et al. [22], 2010 (UK)	Case–control	All specialties of all grades	1999–2004	Medical regulatory authority (Medical Council)	Disciplinary action	Univariate and multivariate analysis adjusted for social class, failed exams in early/preclinical course

^a^ Excluded dermatologists and physical medicine doctors

^b^ Excluded pathologists, radiologists, anaesthesiologists, emergency medicine doctors, and those doctors in administrative and research positions

### Disciplinary action by a medical regulatory body

Disciplinary action was measured by 15/32 studies [5–9, 13–22]. Overall, 12/15 studies found male doctors were more likely to be subject to disciplinary action [6, 7, 13, 14, 16–22]. In 10 of those studies, the sex difference was statistically significant (*P* ≤0.05) [5–7, 16, 17, 19–22], whereas the remaining two did not use inferential statistics [9, 14]. Finally, 3/15 studies found no statistically significant effect of sex [8, 9, 15].

### Malpractice experience

Malpractice experience was reported by 9/32 studies [23–31], of which 6 studies found male doctors were significantly more likely to have malpractice experience than female doctors (*P* ≤0.05) [23, 25–28, 30]. One study examined doctors at two time points and found male doctors were more likely to have malpractice experience in 1991 (*P* = 0.043) but not in 2005 (*P* = 0.168) [31]. The remaining 2/9 studies found no statistically significant association between sex and malpractice [24, 29].

### Referral to a medical regulatory body

Overall, 2/32 studies examined referrals to a medical regulatory body [32, 33]. Both found male doctors were more likely to be referred to a medical regulatory body, with one demonstrating a highly statistically significant association (*P* <0.001).

### Medico-legal matter with a medical defence organisation

In total, 2/32 studies examined medico-legal matters with a medical defence organisation [34, 35]. The association between male sex and medico-legal matter was highly statistically significant (*P* ≤0.005).

### Criminal cases

Criminal cases were examined in 2/32 studies [36, 37]; both found that male doctors were significantly more likely to experience criminal charges (*P* <0.05).

### Complaint to a health care complaints body

Complaints received by a health care complaints body were examined by 2/32 studies [38, 39]; both found that male doctors were more likely to receive complaints. One found a statistically significant effect (*P* <0.05), the other did not provide any inferential statistics.

### Summary of findings

Overall, 27/32 studies found that male doctors were more likely to have had experienced at least one medico-legal action [5–7, 13, 14, 16–19, 21–23, 25–28, 30, 32–39], although 4/27 studies did not calculate inferential statistics and did not provide sufficient data to enable the calculation of any effect size [13, 18, 32, 38]. Of the studies that provided an effect size or where it was possible to calculate an effect size from the data reported, 22/23 demonstrated that male doctors were statistically significantly more likely to have had experienced a medico-legal action (*P* ≤0.05) [5–7, 14, 16, 17, 19–23, 25–28, 30, 31, 33–37, 39]. The remaining study examined doctors at two separate time intervals finding a significant association at the early time point only [31].

Finally, 5/32 studies found no statistically significant difference between male and female doctors [8, 9, 15, 24, 29].

### Assessment of the methodological quality

Methodological quality was assessed using the Newcastle-Ottawa scale [12] (Table 2). Overall, the cohort and case–control studies did not show major problems of selection bias. The main area of weakness for the cohort studies was failing to control for potential confounders. In the case–control studies, the area of weaknesses centred around non-response rates, with 4/7 studies having different rates of response between the controls and the cases [6, 8, 17, 22], and only one describing the non-respondents [17]. The cross-sectional studies varied in methodological quality. The potential for selection bias was present in 3/8 studies [23, 34, 35] and 2/8 studies did not adjust for potential confounders [21, 34]. Ascertainment of exposure and non-response rate was an area of concern in 6/8 studies [23, 25, 29, 31, 34, 35].

**Table 2:** Methodological quality assessment using the Newcastle-Ottawa scale [12]

Cohort studies								
	Selection				Comparability	Outcome		
	Representativeness of exposed cohort	Selection of non exposed cohort	Ascertainment of exposure	Demonstration outcome not present at start of study	Comparability of cohorts	Ascertainment of outcome	Follow-up long enough for outcomes to occur	Adequacy of follow up of cohorts
Alam et al. [14] (2011)	★	★	★	★		★	★	★
Alam et al. [13] (2013)	★	★	★	★		★	★	★
Baldwin et al. [24] (1991)	★	★	★	★	★★	★	★	★
Birkeland et al. [15] (2013)	★	★	★	★	★★	★	★	★
Bismark et al. [38] (2013)	★	★	★	★	★★	★	★	★
Donaldson et al. [39] (2014)	★	★	★	★		★	★	★
Elkin et al. [18] (2011)	★	★	★	★	★	★	★	★
Ely et al. [26] (1999)	★	★	★	★	★★	★	★	★
Goldenbaum et al. [36] (2008)	★	★	★	★		★	★	★
Hickson et al. [27] (2002)	★	★	★	★	★★	★	★	★
Khaliq et al. [19] (2005)	★	★	★	★	★★	★	★	★
Pande et al. [37] (2013)	★	★	★	★		★	★	★
Papadakis et al. [20] (2008)	★	★	★	★	★★	★	★	★
St George[32] (2003)	★	★	★	★		★	★	★
Tamblyn et al. [33] (2007)	★	★	★	★	★★	★	★	★
Taragin et al. [28] (1992)	★	★	★	★	★	★	★	★
Weycker et al. [30] (2000)	★	★	★	★	★★	★	★	★
Case-control and Cross-sectional studies								
	Selection				Comparability	Exposure		
	Case definition	Representativeness of cases	Selection of controls	Definition of controls	Comparability of cases and controls	Ascertainment of exposure	Method of ascertainment for cases and controls	Non-response rate
Cardarelli et al. [16] (2004)	★	★	★	★	★★	★	★	★
Clay et al. [17] (2003)	★	★	★	★	★★	★	★	
Kohatsu et al. [6] (2004)	★	★	★	★	★★	★	★	
Morrison et al. [7] (1998)	★	★	★	★	★★	★	★	★
Papadakis et al. [8] (2004)	★	★	★	★	★★	★	★	
Papadakis et al. [9] (2005)	★	★	★	★	★★	★	★	★
Yates et al. [22] (2010)	★	★	★	★	★★	★	★	
Balch et al. [23] (2011)			★	★	★★		★	
Chauhan et al. [25] (2005)		★	★	★	★★		★	
Nash et al. [35] (2009)			★	★	★★		★	
Nash et al. [34] (2009)	★		★	★			★	
Unwin et al. [5] (2014)	★	★	★	★	★★	★	★	★
Wakeford et al. [21] (2011)	★	★	★	★		★	★	★
Weisman et al. [29] (1988)		★	★	★	★★		★	
Wu et al. [31] (2009)		★	★	★	★★		★	

### Meta-analysis

Of the 32 studies included in the systematic review, 19 reported data that allowed the calculation of a measure of effect and were included in the meta-analysis [5, 6, 8, 9, 14, 16, 19, 20, 22–25, 28, 31, 34–37, 39]. The meta-analysis included 3,794,486 study participants (both cases and non-cases), of which 20,666 are cases of medico-legal action.

### Summary effect estimates

**Fig. 2:**
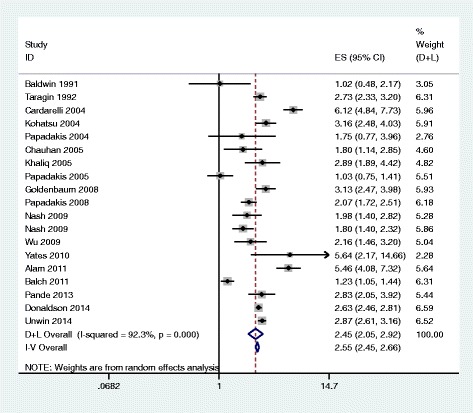
Results of meta-analysis of 19 reports on the association between doctor’s sex and experience of medico-legal action using both fixed-effects and random-effects modelling

A random-effects model found a pooled odds ratio of 2.45 (95 % CI, 2.05–2.93). All 19 studies reported that male doctors were more likely to experience a medico-legal action than female doctors (range of odds ratios, 1.02–6.12) [5, 6, 14, 16, 19, 20, 22, 23, 25, 28, 31, 34–37, 39]; in 3/19 studies the difference was not statistically significant [8, 9, 24]. No studies showed women were more likely to experience a medico-legal action than men (Fig. 2). A high degree of heterogeneity was present (*Q* = 233.25, d.f. = 18, *P* <0.001; *I*^2^ = 92.3 %) which was due to differences in the size rather than the direction of effect.

### Subgroup analyses

#### Study design (cross-sectional, cohort, case–control)

There was a significant overall effect of sex in each stratum, with male doctors having increased odds of medico-legal action (cohort studies: OR, 2.79; 95 % CI, 2.33–3.34; case–control studies: OR, 2.84; 95 % CI, 1.34–6.01; cross-sectional studies: OR, 1.91; 95 % CI, 1.32–2.78). There was high heterogeneity within each stratum (cohort studies: *I*^2^, 81.9 %; case–control studies: *I*^2^, 95.3 %; cross-sectional studies: *I*^2^, 94.1 %).

#### Continent (North America, Europe, Asia, and Australia)

There was a significant overall effect of sex in each stratum, with male doctors having increased odds of medico-legal action (North America: OR, 2.40; 95 % CI, 1.75–3.30; Asia and Australia: OR, 1.92; 95 % CI, 1.60–2.30; Europe: OR, 2.76; 95 % CI, 2.48–3.07). There was high heterogeneity in the North America stratum (*I*^2^, 94.5 %). The degree of heterogeneity was small for both Europe (*I*^2^, 55.1 %) and Asia and Australia (*I*^2^, 0.0 %), but these strata only contained three studies each and therefore we are not able to accurately estimate heterogeneity.

#### Outcome (disciplinary action, malpractice, other)

Male doctors were significantly more likely to have experienced a medico-legal action in all three strata (disciplinary action: OR, 2.95; 95 % CI, 2.12–4.10; malpractice: OR, 1.74; 95 % CI, 1.11–2.71; other: OR, 2.46; 95 % CI, 2.05–2.94). There was considerable heterogeneity present in the strata disciplinary action (*I*^2^, 93.2 %) and malpractice experience (*I*^*2*^, 92.2 %).

#### Year from which data was collected

Male doctors were significantly more likely to have experienced a medico-legal action in all strata, apart from in 1985–1989, where the result was not statistically significant (1985–89: OR, 1.79; 95 % CI, 0.69–4.65; 1995–99: OR, 6.12; 95 % CI, 4.84–7.73; 2000–04: OR, 2.37; 95 % CI, 1.31–4.28; 2005–09: OR, 2.45; 95 % CI, 1.81–3.31; 2010–14: OR, 2.25; 95 % OR, 1.63–3.12). There was substantial heterogeneity throughout all the strata.

#### Specialty and Grade (all specialties, individual specialties)

Although we had planned to conduct subgroup analyses examining grade and specialty, we decided not proceed because the data available did not allow the formation of meaningful groups.

### Bias

We used a funnel plot to assess possible bias (Additional file 1: Figure S1). The distribution of the studies within the funnel plot did appear somewhat random with eight of the studies appearing outside of the funnel. This suggests heterogeneity in the studies. There was no sign of a publication bias relating to the significance of the effect found. There was a sparsity of studies at the bottom of the plot, with the majority of the studies clustering towards the top of the graph. The relative absence of studies towards the bottom of the graph could indicate an absence of smaller studies. This may reflect the nature of the data typically available to allow such studies.

## Discussion

### Summary of main results

Male doctors had nearly 2.5 times the odds (pooled OR, 2.45; 95 % CI, 2.05–2.93) of medico-legal action compared to female doctors. There was significant heterogeneity in the meta-analysis but this was not due to differences in the direction of the effects – no studies found that women were more likely than men to experience medico-legal action. The size of the effect of sex on experience of medico-legal action remained roughly constant in all subgroup analyses, suggesting that the effect of sex is not influenced by the study design, the country the doctor is in employed in, or the outcome definition, and the effect seems stable over time.

### Overall completeness and applicability

#### Literature search

The literature searching was thorough, as demonstrated by the number of reports initially identified (>6,500). We did not extensively search grey literature sources due to limited resources and the vast number of reports obtained with the search methods used. It is possible that smaller studies, or studies that did not demonstrate a sex effect, may have been overlooked. Another limitation was the exclusion of studies whose abstract and/or full-text were not available in English or French – seven studies judged to be potentially eligible based on their titles were excluded for this reason [40–46]. The exclusion of studies not available in English may partly explain why the majority of the studies included in this report are from English-speaking, high-income countries. It is possible that literature from non-English speaking countries demonstrate a different size of effect of doctors’ sex on experience of medico-legal action, and as such the results of this report may not be applicable to non-English speaking countries.

#### Study selection

In our systematic review, we were able to capture >40,000 cases of medico-legal action against doctors, capturing >20,500 medico-legal action cases in the meta-analysis. These large numbers of cases allowed meaningful conclusions to be drawn from the results. The majority of the studies attempted to collect data applicable to the wider population (country-wide, state-wide, etc.), a likely reflection that a doctor experiencing a medico-legal action is a relatively rare outcome, and therefore large studies are required to attempt to capture as many cases as possible. Capturing data from the wider population does mean that the results are more likely to be generalizable. Our reported studies covered eight countries over four continents. We demonstrated that, when stratifying the data by continent, the pooled results for each stratum was relatively stable, with male doctors having approximately two times the odds of experiencing a medico-legal action. There was variability in the effect size within the North America stratum, though the direction of the effect remained consistent.

It is worth mentioning that, of the three studies included in the Asia and Australia stratum, two were from Australia and one was from Taiwan, and although the heterogeneity was small in this stratum, it did only include three studies. Within the Europe stratum, all three studies were from the UK, and therefore the stratum may not be an accurate reflection of Europe. The total number of studies and the limited range of countries from where the studies were from in both the Asia and Australia stratum and in the Europe stratum highlight the limitation of only including studies which were published in English or French.

The outcome definition used by the individual studies varied in severity. We chose to use a variety of terms to capture the outcome, with the aim of capturing as many relevant studies as possible. Because the different outcome types varied in severity, it may not be fair to include studies together. That said, the sub-analysis examining the data by outcome type showed that the overall effect of doctors’ sex was consistent, with male doctors having approximately 2–3 times the odds of experiencing a medico-legal action in each stratum. It is also interesting to note that the two largest strata were the outcomes ‘disciplinary action’ and ‘malpractice’, both of which have severe impact on a doctor’s professional career.

The demographics of doctors in the UK and USA have been changing, with increasing numbers of women choosing to follow medicine as a career [47, 48]. Our results suggest that the effect of male sex on experiencing medico-legal actions has remained fairly constant over the last 15 years (OR, 2.25–2.45), despite the increasing trend of women doctors (it is not possible to comment on the years prior to 2000 due to the small number of studies in the strata). We therefore feel one can no longer argue that male doctors are more likely to face medico-legal action because there are more male doctors practising. If this were the case, we would expect the effect size to diminish over time, to reflect the increasing number of female doctors.

Unfortunately, we were unable to explore further whether the sex difference in medico-legal action was impacted by specialty practised. Thirteen of the studies included in the meta-analysis examined whether the likelihood of medico-legal action differed between specialties [5, 6, 8, 9, 14, 16, 19, 28, 31, 35–37, 39]; however, the specialities most and least likely to face medico-legal action varied greatly between the studies. In the studies which controlled for the effect of specialty when examining the association between sex and medico-legal action, all but one [31] demonstrated that male doctors remained more likely to have had medico-legal experience even with specialty taken into account [6, 5, 16, 19, 35].

Other variables have been shown to be both associated with doctors’ sex and experience of medico-legal action, but have not been examined by this meta-analysis. These include the number of hours worked or the number of patient encounters. Studies included in this systematic review and meta-analysis have demonstrated that female doctors work less hours than male doctors [34, 35] and see less patients than their male colleagues [28]. The number of hours worked has been shown to be associated with increased likelihood of medico-legal action in three of these studies [23, 34, 35]. Exploring how the number of hours worked or number of patient encounters differ between the sexes and the effect on medico-legal action may be of interest for a future review of the literature to help towards understanding the sex difference in medico-legal action.

### Other potential biases in the review process

One reviewer assessed the reports for eligibility, and this was not performed blind – this could have introduced bias; however, the reviewer did use previously agreed criteria to guide their decisions, with the aim of reducing bias. Another possible source of bias is that the outcome definitions used were not wide enough, and that there may be some culturally specific terms that the researchers, who are all from the UK, were unaware of. Finally, the assessment of the methodological quality of the studies is subjective. To reduce this source of bias, two researchers independently judged the methodological quality of the studies and the Newcastle-Ottawa scale checklist was used to guide and support our decisions. We also chose not to exclude any studies from the systematic review or meta-analysis based on the findings of the appraisal of methodological quality.

## Conclusions

This is the first systematic review and meta-analysis examining the association between doctors’ sex and experience of a medico-legal action. It demonstrates that male doctors are more likely to have had experience of a medico-legal action when compared to female doctors. This effect was demonstrated over a number of years, across a range of study designs, across different countries, and with a wide definition of outcome types, and therefore seems robust. The demonstration of a consistent effect size, present in the main analysis, as well as in the subgroup analyses, highlights that there is likely to be a fundamental reason to explain why male doctors are at over two times the odds of experiencing a medico-legal action.

More detailed information is needed to understand the reasons why male doctors are more likely to experience a medico-legal action. The causes are likely to be complex and multi-factorial, but the first step is to recognise that there is a difference, and this study shows that robustly. Medical schools, medical regulatory authorities, and researchers now need to work together to try to further understand the difference between the sexes that could explain the difference in experience of medico-legal action, with the aim of better supporting our doctors and improving patient safety.

## Additional file

Additional file 1: Table S1. Search terms used for electronic database search. **Table S2.** Names of journals and conference abstracts searched electronically. **Figure S1.** Funnel plot of effect size by inverse standard error.

## Abbreviations

CI: Confidence interval
GMC: General Medical Council
OR: Odds ratio
UK: United Kingdom
