# Supplementary material for: Sex differences in medico-legal action against doctors: a systematic review and meta-analysis
Source: BMC Med. 2015 Aug 13;13:172. doi: 10.1186/s12916-015-0413-5 (PMC4535538; doi:10.1186/s12916-015-0413-5)

Table S1: Search terms used for electronic database search

MEDLINE search in January 2015

Concept 1	Concept 2	Concept 3
MeSH term Physicians<br>Doctor?<br>Medical Practitioner?<br>Medic?<br>Clinician?	MeSH term Gender Identity<br>MeSH term Male<br>MeSH term Female<br>(Male and Female MeSH terms combined with AND)<br>Sex	MeSH term Malpractice<br>MeSH term Employee Performance Appraisal<br>Clinical competen*<br>Disciplinary board<br>Disciplinary action<br>Professional misconduct<br>Fitness to practi*<br>Medical regulat*

Embase search conducted in January 2015

Concept 1	Concept 2	Concept 3
MeSH term Physician<br>Doctor?<br>Medical Practitioner?<br>Medic?<br>Clinician?	MeSH term Gender and Sex<br>MeSH term Gender Identity<br>MeSH term Gender<br>MeSH Male<br>MeSH term Female<br>(Male and Female MeSH terms combined with AND)<br>MeSH term Sex	MeSH term Malpractice<br>MeSH term Job Performance<br>MeSH term Performance<br>MeSH term Professional Practice<br>MeSH term Professional Misconduct<br>Clinical competen*<br>Disciplinary board<br>Disciplinary action<br>Fitness to practi*<br>Medical regulat*<br>Professional performance

PsycINFO search conducted in January 2015

Concept 1	Concept 2	Concept 3
MeSH term Physicians<br>MeSH term Family Physicians<br>Doctor?<br>Medical Practitioner?<br>Medic?<br>Clinician?	MeSH term Gender Identity<br>Sex	MeSH term Professional Liability<br>MeSH term Professional Competence<br>Clinical competen*<br>Disciplinary board<br>Disciplinary action<br>Professional misconduct<br>Fitness to practi*<br>Medical regulat*<br>Professional performance

Table S2: Names of journals and conference abstracts searched electronically

Journals that yielded the majority of eligible articles
BMJ Quality & Safety
British Medical Journal
The American Journal of Medicine
The Journal of the American Medical Association
The Medical Journal of Australia
**Journals felt to be important in the medical education field**
Academic Medicine
Medical Education
Medical Teacher
The New England Journal of Medicine
**Medical education conference abstracts**
The Association for Medical Education in Europe
The Association for the Study of Medical Education

Figure S1: Funnel plot of effect size by inverse standard error

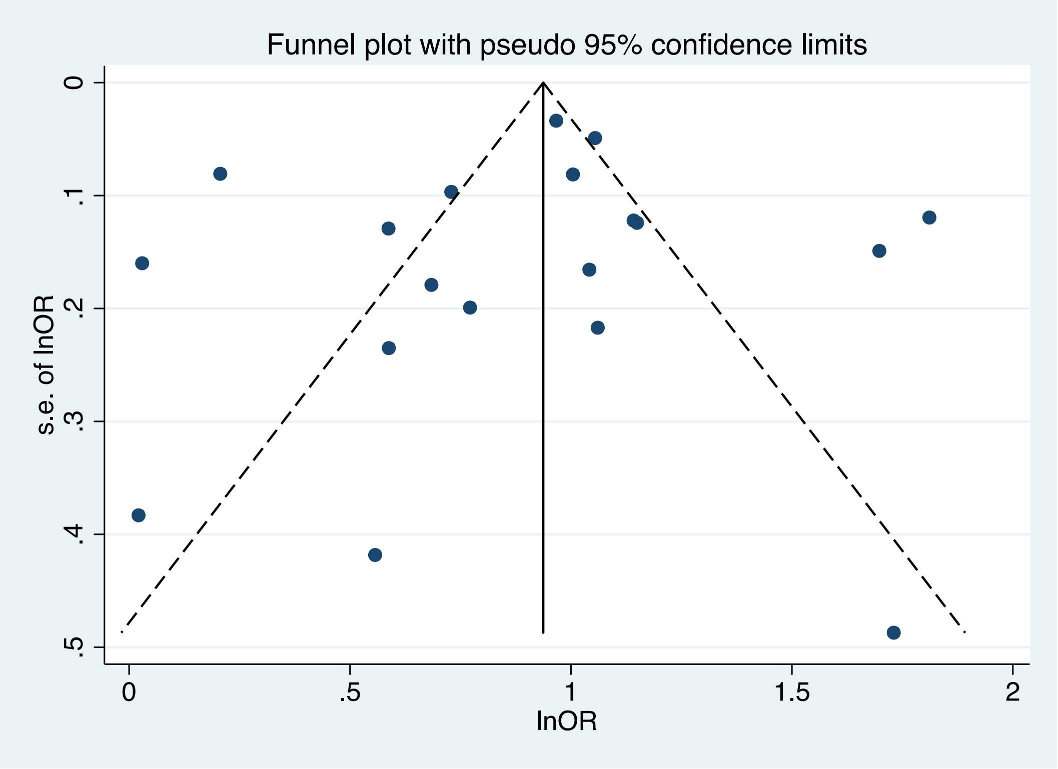

Supplement: Additional file 1: Table S1. — Search terms used for electronic database search. Table S2. Names of journals and conference abstracts searched electronically. Figure S1. Funnel plot of effect size by inverse standard error. [file 12916_2015_413_MOESM1_ESM.pdf]
